# Flavonoid Group of *Smilax glabra* Roxb. Regulates the Anti-Tumor Immune Response Through the STAT3/HIF-1 Signaling Pathway

**DOI:** 10.3389/fphar.2022.918975

**Published:** 2022-07-22

**Authors:** Yingxue Guo, Weiye Mao, Lu Jin, Linying Xia, Jie Huang, Xia Liu, Ping Ni, Qiyang Shou, Huiying Fu

**Affiliations:** ^1^ The Second Clinical Medical College of Zhejiang Chinese Medical University, Hangzhou, China; ^2^ School of Pharmaceutical Sciences, Zhejiang Chinese Medical University, Hangzhou, China; ^3^ Zhejiang Provincial Key Laboratory of Sexual Function of Integrated Traditional Chinese and Western Medicine, Hangzhou, China; ^4^ School of Basic Medical Sciences, Zhejiang Chinese Medical University, Hangzhou, China; ^5^ College of Pharmacy, Hubei University of Traditional Chinese Medicine, Wuhan, China

**Keywords:** *Smilax glabra* Roxb., STAT3/HIF-1 signaling pathway, flavonoid, M1 phenotype, tumor-associated macrophages

## Abstract

**Background:** *Smilax glabra* Roxb. (SGR) is a widely used traditional Chinese medicine, which has known effects of enhancing immunity. However, its anti-tumor effects and mechanism of action are still unclear.

**Methods:** We selected MMTV-PyMT mice to determine the anti-tumor efficacy of SGR ethyl acetate (SGR-EA). First, flow cytometry was used to detect the number of immune cells in the mice tumor microenvironment. Furthermore, M2 polarization of macrophages was stimulated *in vitro*, and the expressions of macrophage M1/M2 surface markers and mRNA were as determined. Finally, we carried out a network pharmacology analysis on the active components of SGR-EA and *in vitro* experiments to verify that SGR-EA regulated the hypoxia-inducible factor (HIF)-1 signaling pathway to modulate the anti-tumor immune response by resetting M2 macrophages toward the M1 phenotype which inhibited tumor growth and lung metastasis in the mice.

**Result:** SGR-EA inhibited tumor growth and lung metastasis in the mice. Tumor-associated macrophages switched from M2 to the tumor-killing M1 phenotype and promoted the recruitment of CD4^+^ and CD8^+^ T cells in the tumor microenvironment. *In vitro*, SGR-EA significantly inhibited the polarization of macrophages into M2 macrophages and increased the number of M1 macrophages. In addition, following an intervention with SGR-EA, the expression of the HIF-1 signaling pathway-related proteins stimulated by interleukin-4 in macrophages was significantly inhibited.

**Conclusion:** SGR-EA played an anti-tumor role by inhibiting the activation of the HIF-1 signaling pathway and response by resetting tumor-associated macrophages toward the M1 phenotype.

## Introduction

In recent years, tumor immunotherapy has brought about great innovation in the treatment of various cancers such as breast cancer and prostate cancer (Todoric et al., 2016; Mulder and Gnjatic, 2017). However, the immunosuppressive tumor microenvironment (TME) remains a key factor impeding tumor immunotherapy (Donahue et al., 2015). So far, most studies to date have focused on macrophages, which are the primary components of numerous immune cells in the TME. Tumor-associated macrophages (TAMs) are a general term for macrophages that infiltrate tumor tissues, and have been proven to be closely related to tumor growth, angiogenesis, metastasis, and tumor immune escape (Wenes et al., 2016; Wei et al., 2019). Therefore, TAMs are considered to be one of the potential targets for anti-tumor drugs. Although TAMs has pro-tumor effects in most cases, it can also kill tumor cells by releasing tumor necrosis factor-α (TNF-α) and interferon-γ (IFN-γ) to promote the immune response of Th1 (helper cells) (Mantovani et al., 2017). Notably, TAMs have the same phenotype as M2 macrophages, which are alternately activated by Th2 cytokines interleukin-4 (IL-4), interleukin-13 (IL-13), and others. In contrast, M1 macrophages are capable of killing tumor cells and resisting pathogen invasion, and are usually activated by Th1 cytokines such as IFN-γ (Wu et al., 2020). Therefore, an ideal way to target TAMs is to convert M2 TAMs to M1 anti-tumor macrophages rather than depleting them.

*Smilax glabra* Roxb. (SGR) is the dried rhizome of *Smilax glabra*, a Liliaceae plant, widely used to treat syphilis, gout, gonorrhea, inflammation, and cancer (Zou et al., 2017). Recent studies have shown that an SGR extract can exert anti-tumor effects by inducing mitochondrial apoptosis in tumor cells, and inhibit the proliferation of HepG2, HT-29, MCF-7, BGC-823, and other tumor cells in a dose-dependent manner (Samarakoon et al., 2012; Gao et al., 2018). However, there is still a lack of scientific anti-tumor evaluations and mechanistic explorations. Notably, *in vitro* immune regulation experiments showed that the SGR extract could activate macrophages and enhance the phagocytosis of macrophages, and increase the secretion of NO, IL-6 and TNF-α (Wang et al., 2017). This suggests that SGR has the potential to be developed as a novel immunomodulator for tumor immunotherapy, because an effective tumor immunotherapy requires the participation of macrophages.

In this study, we selected the MMTV-PyMT (FVB) mouse model to evaluate the inhibitory effect of the SGR ethyl acetate (SGR-EA) extract on tumor growth and metastasis. The changes were analyzed in macrophage subsets in the TME *in vivo*, as well as *in vitro*. We also analyzed and verified the anti-tumor mechanism of SGE-EA through network pharmacology. Our results show that SGR-EA, as an immunomodulator, can adjust its anti-tumor efficacy by resetting TAMs from the M2 phenotype to the M1 phenotype.

## Materials and Methods

### Preparation and Properties of Total Flavonoids Obtained From *Smilax glabra* Roxb

SGR was purchased from Pan’an County, Zhejiang Province, and the batch number production is 20142907. It was identified by Professor Chen Hongshu of the TCM pharmacy of Zhejiang Hospital of Traditional Chinese Medicine. Voucher specimens are stored in the specimen room of the Second Clinical Medical College of Zhejiang China Medical University for future repetitive research. The samples were pulverized and extracted with 75% ethanol at a solid–liquid ratio of 1:10 (W/V) thrice, each for 1 h, and then concentrated under reduced pressure at 40°C to obtain the total alcohol extract. Then, petroleum ether and ethyl acetate were used to extract the total alcohol extract, i.e., the ethyl acetate fraction (SGR-EA) (Chao et al., 2020; Kwon et al., 2020). The solvent in the fraction was removed under vacuum, and the residue was freeze-dried and stored at 4°C until further use.

The components of SGR-EA were determined by ultra-high-performance liquid chromatography–mass spectrometry (UPLC-Q/TOF MS). SGR-EA was dissolved in methanol and filtered, and the supernatant was collected. The following chromatographic conditions were set: Waters SYNAPT G2-SI UHPLC system and a CORTECS UPLC T3 column (100 × 2.1 mm, 1.6 μm, Waters, Milford, MA, United States) at 35°C. The mobile phases were solvent A (0.1% formic acid aqueous solution, chromatographic grade, Merck) and solvent B (acetonitrile, chromatographic grade, Merck). The injection volume was 2 μl, and the flow rate was 0.3 ml/min for, 35 min in total. The flow rate gradient was set at 0–2 min, 5% B; 2–32 min, 5–100% B; 32–33 min, 100% B; 33–33.5 min, B 100%–5%; and 33.5–35 min, 5% B. The ion source parameters were set as follows: anion capillary voltage, 2500 V; ion source temperature, 120°C, dissolvent temperature, 400°C, dissolvent gas flow rate, 800 L/h; and atomizing gas pressure, 6.0 bar. Data acquisition and analysis were controlled using the Waters MassLynx 4.1 software. The molecular formulas and weights of the compounds were defined in the Unififi 2.0 software (Waters, Milford, MA, United States) database. Meanwhile, we obtained the structure of the compound from PubChem.

### Animal Experiments

Female MMTV-PyMT (FVB) and C57BL/6J mice were obtained from the Jackson Laboratory. This experiment was approved by the Ethics Review Committee of the Laboratory Animal Center of Zhejiang University of Traditional Chinese Medicine (approval no: IACUC-20201019-03). Seven-week-old female MMTV-PyMT mice (19–21 g) were selected and randomly divided into a model group and a SGR-EA group. The animals were intragastrically administered SGR-EA (0.4 g/kg) and 0.5% carboxyl methyl cellulose every day for 44 days. The volume of each palpable tumor nodule (>20 mm^3^) was measured weekly, and the tumor volume was calculated as V = (length × width^2^)/2. The mice were euthanized with CO_2_ on day 45, and tumor and lung tissues were collected. The tumors were then removed, weighed, cut, and sectioned into three parts. The largest lump and lung were fixed with 4% paraformaldehyde for histopathological and immunohistochemical examinations. The tumor tissue was ground, the immune microenvironment was evaluated by flow cytometry, and the remaining tissues were frozen at −80°C.

### Hematoxylin and Eosin Staining of Lung and Tumor Tissues

The fixed lung and tumor tissues were taken, dehydrated until transparent, and after embedding paraffin, cut it into 4 μm slices. The sections were stained with H and E. The slices were imaged using a NanoZomer digital slice scanner (Nikon, Tokyo, Japan), and the lung metastasis area was assessed using the NDP.view2U12388-01 digital pathology scanning software (Hamamatsu, Japan).

### Flow Cytometry Analysis

To assess the immune cell invasion of the TME, all samples were collected, processed, and stained, as described previously (Jin et al., 2020). In brief, after washing twice with a fluorescence-activated cell sorting (FACS) buffer (phosphate-buffered saline, 2% heat-inactivated fetal calf serum, 1% penicillin-streptomycin solution, and 0.1% sodium azide), the tumors were placed in a harvest medium and ground to form a single-cell suspension. Cells were then placed on ice in an FACS buffer and stained for extracellular T cells and myeloid-derived cell antibody panels. All antibodies used for flow cytometry staining were purchased from Bioscience, Horizon, or BD. Details are as follows: CD45 FITC, Anti-CD4 PE, anti-CD8a APC-H7, anti-F4/80 PE, anti-CD11b AF700, anti-CD206 AF647, and anti-CD86 BV510. Data for both antibody groups were obtained by FACS Canto II Cytometry (BD Biosciences) flow cytometry and analyzed.

### Targeting Genes and Bioinformatics Analysis of *Smilax glabra* Roxb

To further clarify the pathway through which SGR-EA inhibits breast cancer progression, SwissTargetPrediction was used to predict potential targets of the main components of SGR-EA, and Cytoscape (3.7.1) was used to construct the component–target network of SGR-EA. Thereafter, all the potential targets were collected, Gene Ontology (GO) and Kyoto Encyclopedia of Genes and Genomes (KEGG) enrichment analyses were performed with R package (4.0.2), and a pathway correlation analysis of the potential targets was performed using ClueGO. Finally, we used STRING and constructed the protein–protein interaction network, identified the top five key genes by Cytohubba, and visualized them using Cytoscape.

### Murine Peritoneal Macrophages

As previously described (Fu et al., 2020), thioglycolic acid-induced murine peritoneal macrophages (PMs) were prepared from female C57BL/6 J mice aged 6–8 weeks. PMs were cultured in Dulbecco’s Modified Eagle Medium (Gibco, United States) supplemented with 10% fetal bovine serum (Gibco, United States) and 2% 100× penicillin–streptomycin solution, (Biosharp, China Guangzhou). The cells were then seeded in a 12-well plate at a density of 1×10^6^ cells/well. After 24 h, the adherent cells were primary PMs. The cells were treated with either SGR-EA or IL-4 (20 ng/ml) at cell-sensing concentrations of 100 and 200 μg/ml, and collected 24 and 48 h later for quantitative real-time PCR (qRT-PCR), FACS, and Western blot assays, respectively.

### Real-Time Cellular Analysis

First, 50 µl of the culture medium was added to the wells of the e-plate 96. After baseline testing, 100 µl was added and mixed evenly, and then, PM cell suspension was added to each well so that the number of cells in each was 2 × 104 cells. The e-plate 96 was placed on the RTCA station in the incubator. The RTCA program was used to detect the cell index every 15 min. On the second day, 50 µl SGR-EA was added to the well of an e-plate 96 to adjust the drug concentration to 800 µg/ml, 400 µg/ml, 200 µg/ml, and 100 µg/ml, and the RTCA program was set to detect the cell index every 2 min. After 72 h, the e-plate 96-well plate was removed, and the data were analyzed using RTCA Software 2.1.

### RNA Extraction and Quantitative Real-Time PCR

Total RNA was isolated from TRIzol by chloroform/ethanol extraction, precipitated, transcribed, and reverse-transcribed into cDNA, according to the manufacturer’s instructions. qRT-PCR was performed using a Bio-Rad CFX-96 touch system (United States) and iCycler technology. qRT-PCR primers were designed by Sangon Biotech, as shown in Table 1. Amplification was performed according to the instructions of the TB Green kit (TaKaRa, Dalian, China), and the data were analyzed by LightCycler^®^ 96 SW 1.1.

**TABLE 1:** Primer sequences of the genes.

No.	Gene	Primer sequence (5′ → 3′)
1	IL-10	F-5′-GGTTGCCAAGCCTTATCGGA
		R-5′-ACCTGCTCCACTGCCTTGCT
2	TNF-α	F-5′-CGAGTGACAAGCCTGTAGCCC
		R-5′-GGGCAGCCTTGTCCCTTGA
3	Arginase 1 (Arg1)	F-5′-CCACAGTCTGGCAGTTGGAAG
		R-5′-GGTTGTCAGGGGAGTGTTGATG
4	IL-1β	F-5′-GAGCACCTTCTTTTCCTTCATCTT
		R-5′-TCACACACCAGCAGGTTATCATC
5	Fizz1	F-5′-TCCCAGTGAATACTGATGAGA
		R-5′-CCACTCTGGATCTCCCAAGA
6	β-Actin	F-5′-GCCATGTACGTAGCCATCCA
		R-5′-GAACCGCTCATTGCCGATAG

### Molecular Docking

To further clarify whether SGR plays a role through the hypoxia-inducible factor (HIF-1) signaling pathway, we conducted molecular docking between the active components of SGR and HIF-1, and the specific operations were as follows: first, the HIF-1 protein structure was obtained from the PDB database, and the chemical structure formula of SGR-active components was obtained from PubChem. Finally, AutoDockTools-1.5.7 was used for the molecular docking analysis.

### Western Blot Analysis

Cell proteins were extracted with a radio-immune precipitation assay buffer containing 100 mM phenylmethylsulfonyl fluoride (Beyotime, Shanghai, China). The proteins were then isolated by SDS-PAGE, transferred to a polyvinylidene difluoride membrane (Millipore, Bedford, United States), and incubated with different antibodies. For example, HIF-1α (Abcam, 1:1,000), STAT3 (ImmunoWay, 1:1,000), and β-actin (Cell Signaling Technology, 1:2,000) were used. The secondary antibody (ImmunoWay, 1:10,000) was then conjugated with horseradish peroxidase. The bands were detected by enhanced chemiluminescence (ProteinSimple, United States). Finally, ImageJ software (1.48v) was used for the semi-quantitative analysis. All experiments were repeated three times independently, with β-actin as the reference gene.

## Results

### Compositional Analysis of *Smilax glabra* Roxb–Ethyl Acetate

**FIGURE 1:**
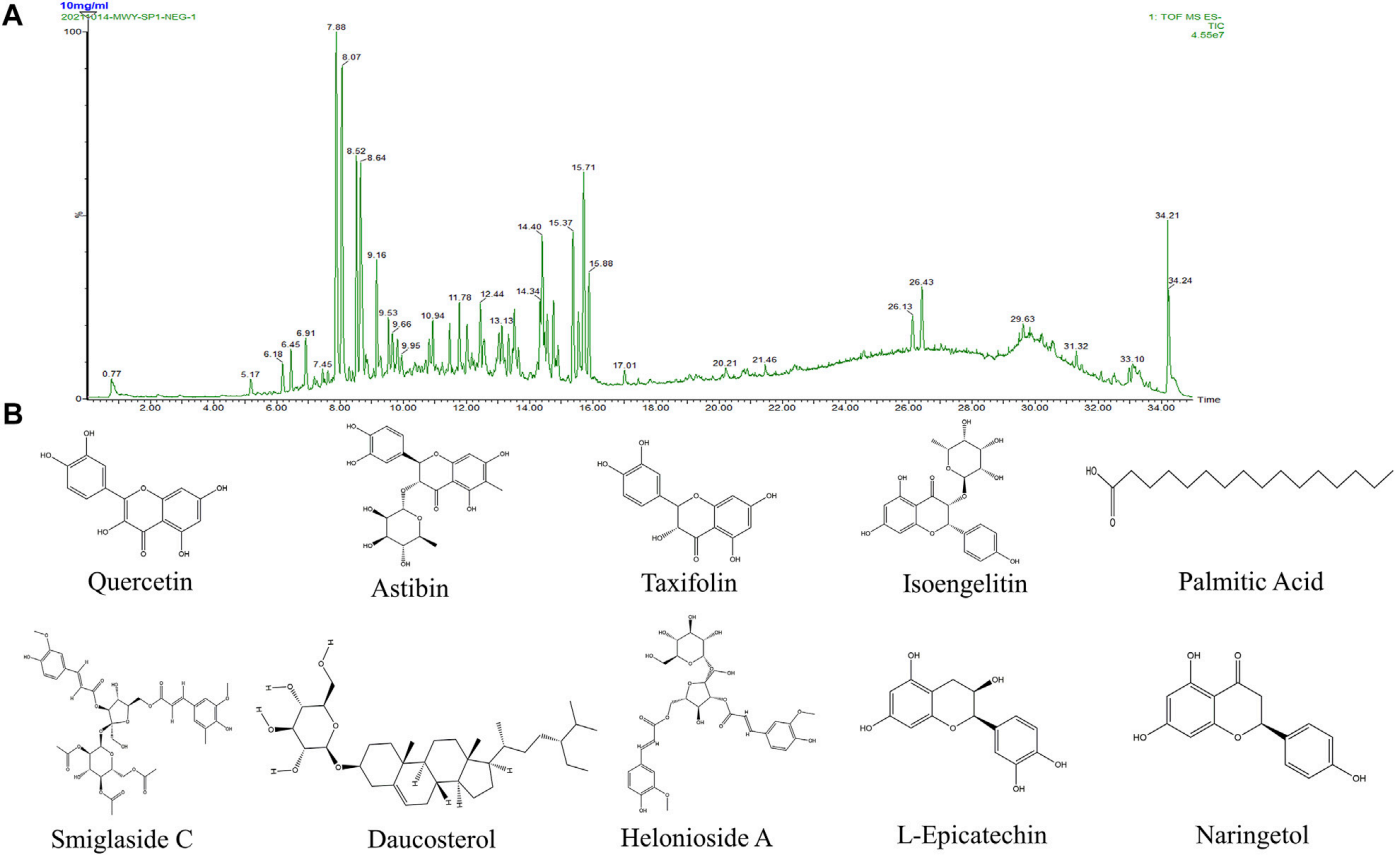
Ethyl acetate layer mass spectrometry of SGR. **(A)** Ultra-performance liquid chromatography of the main components of the *Smilax glabra* Roxb. extract (SGR-EA). **(B)** Chemical structures of the components in the extract (SGR-EA).

The components in SGR-EA were determined by UPLC-Q/TOF MS. The mass spectrum of SGR-EA is shown in Figure 1A, and the results show that SGR-EA comprises of 14 main components: smiglaside D, smiglaside E, and smiglaside A, astilbin, smiglaside B, smiglaside C, daucosterol, helonioside A, L-epicatechin, isoengelitin, hexadecanoic acid (palmitic acid), naringetol, taxifolin, and quercetin (Figure 1B, Table 2).

**TABLE 2:** SGR-EA active substance composition.

Name	Formula	Molecular weight	RT (min)
Smiglaside D	C_47_H_50_O_22_	966.27937	15.72
Smiglaside E	C_45_H_48_O_21_	924.26881	14.4
Smiglaside A	C_48_H_52_O_23_	996.28994	15.89
Astilbin	C_21_H_22_O_11_	450.11621	7.88
Smiglaside B	C_46_H_50_O_22_	954.27937	14.56
Smiglaside C	C_38_H_44_O_20_	820.24259	13.13
Daucosterol	C_35_H_60_O_6_	576.43899	30.58
Helonioside A	C_32_H_38_O_17_	694.2109	9.82
L-Epicatechin	C_15_H_14_O_6_	290.07904	6.45
Isoengelitin	C_21_H_22_O_10_	434.1213	8.84
Hexadecanoic acid	C_16_H_32_O_2_	256.24023	27.38
Naringetol	C_15_H_12_O_5_	272.06847	11.77
Taxifolin	C_15_H_12_O_7_	304.0583	7.88
Quercetin	C_15_H_10_O_7_	302.04265	10.71

### *Smilax glabra* Roxb–Ethyl Acetate Therapy Enhanced the Anticancer Immune Response

**FIGURE 2:**
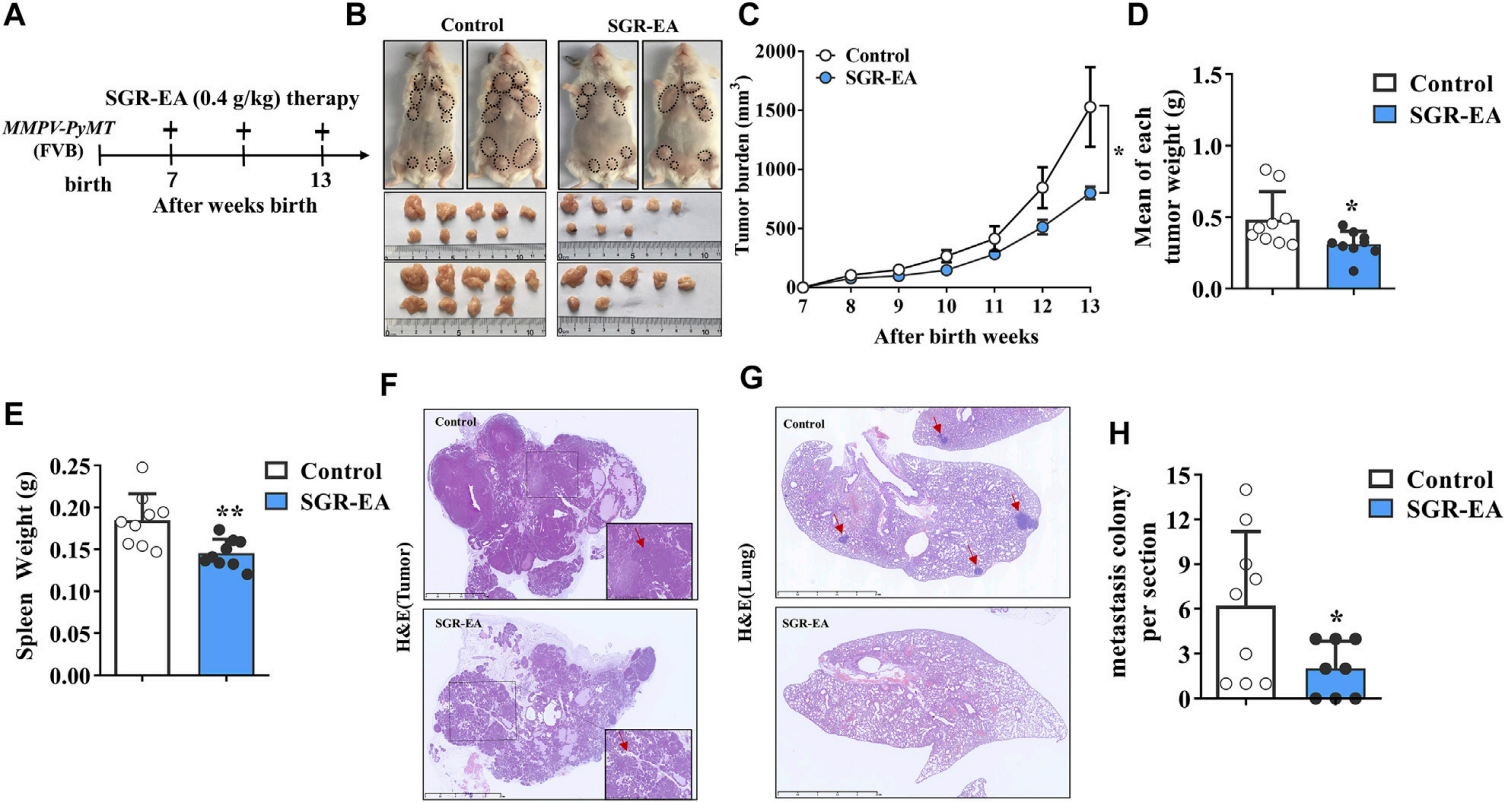
SGR-EA therapy enhances the anti-cancer immune response in MMTV-PyMT mice. **(A)** Diagram depicting the treatment schedule and indicating the continuous daily administration of SGR-EA intragastrically from 7 weeks after birth. **(B)** Representative image showing the gross appearance of tumors. Dotted lines demarcate palpable breast tumor masses. **(C)** Comparison of the total tumor burden. (Tumor burden = the volume of tumor nodules in each mouse was added up.) **(D, E)** Comparison of the mean tumor and spleen weight. (Mean of each tumor weight = Mean weight of total tumor nodules per mouse.) **(F)** H and E–stained tumor sections showing intratumoral regions were viewed under high magnification (lower right panel). Scale bars, 2.5 mm. **(G)** Lung sections stained with H and E were viewed under high magnification. Scale bars, 2.5 mm; the position indicated by the red arrow is the lung metastatic nodule. **(H)** Comparison of the number of metastatic colonies (>100 μm in diameter) per lung section. Unless otherwise denoted, *n* ≥ 8 for each group, and values are mean ± SD. *p < 0.05 and **p < 0.01 versus control.

We evaluated the potential therapeutic effect of SGR-EA in the MMTV-PyMT model when SGR-EA (0.4 g/kg) was administered intragastrically to the mice continuously from 7 weeks after birth. The treatment cycle is shown in a schematic diagram (Figure 2A). When compared with the control group, the SGR-EA group inhibited the total tumor burden in mice from week 10, and the effect was the most significant at week 13 (Figures 2B,C). The mean total tumor and spleen weights in the SGR-EA group were significantly lower (Figures 2D,E). H and E staining showed that in the SGR-EA group, the acinar structure formed early, the invasion was small, and the boundary with the surrounding tissues was obvious. However, in the control group, the invasive ductal carcinoma area extensively invaded the surrounding tissue, and no acinar structure remained (Figure 2F). Furthermore, the number of nodules in the SGR-EA group was much smaller than those in the model group (Figure 2H).

### Improvement of the Tumor Immune Microenvironment by *Smilax glabra* Roxb–Ethyl Acetate

**FIGURE 3:**
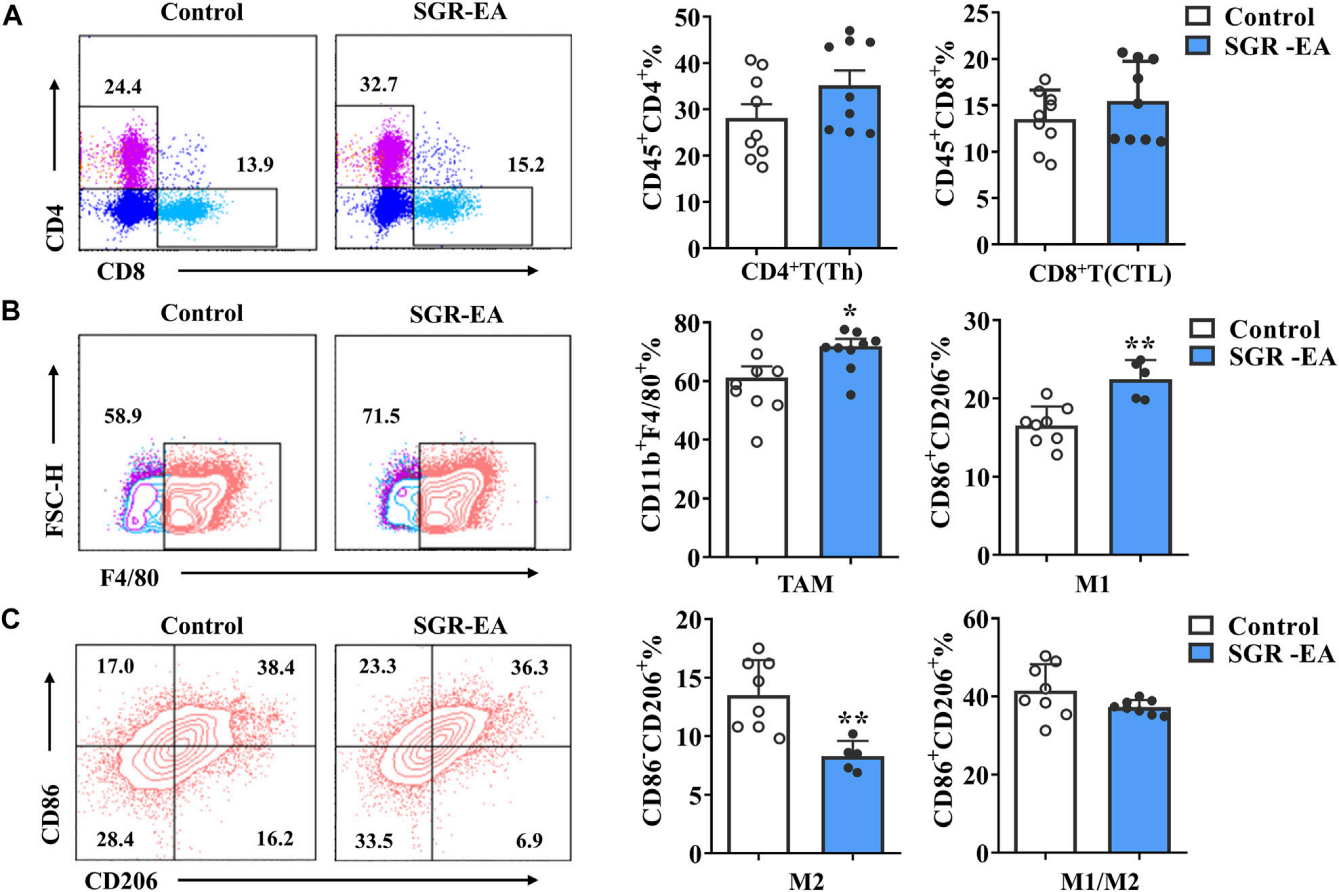
SGR-EA treatment improved the immune microenvironment of breast cancer. **(A)** CD4^+^ T cells (CD45^+^CD4^+^) and CD8^+^ T-cell (CD45^+^CD8^+^) expressions in the tumor. **(B,C)** TAM (CD11b^+^ F4/80^+^) expressions in the tumor; M1 (CD11b^+^ F4/80^+^CD86^+^CD206^−^), M2 (CD11b^+^ F4/80^+^CD86^−^CD206^+^), and M1/M2 (CD11b^+^ F4/80^+^CD86^+^CD206^+^) expressions in the tumor. Unless otherwise denoted, *n* ≥ 5 for each group, and data are presented as means with error bars denoting SD. *p < 0.05 and **p < 0.01 versus control.

To determine whether the effect of SGR-EA on TME can also change immune cell infiltration into the tumor in MMTV-PyMT mice, we collected tumor tissues from all experimental animals and analyzed T-cell and macrophage subsets by flow cytometry. The results showed that, compared with the control group, the SGR-EA group showed promoted infiltration of CD8^+^ T cells and CD4^+^ T cells in the tumor tissue (Figure 3A), but it was not statistically significant. Furthermore, macrophage infiltration was significantly higher in the SGR-EA group than that in the model group. Importantly, TAM cell infiltration was accompanied by an increase in M1 and a decrease of M2, which was confirmed by the presence of CD86^+^CD206^−^ and CD86^−^CD206^+^ cells (Figures 3B,C).

**FIGURE 4:**
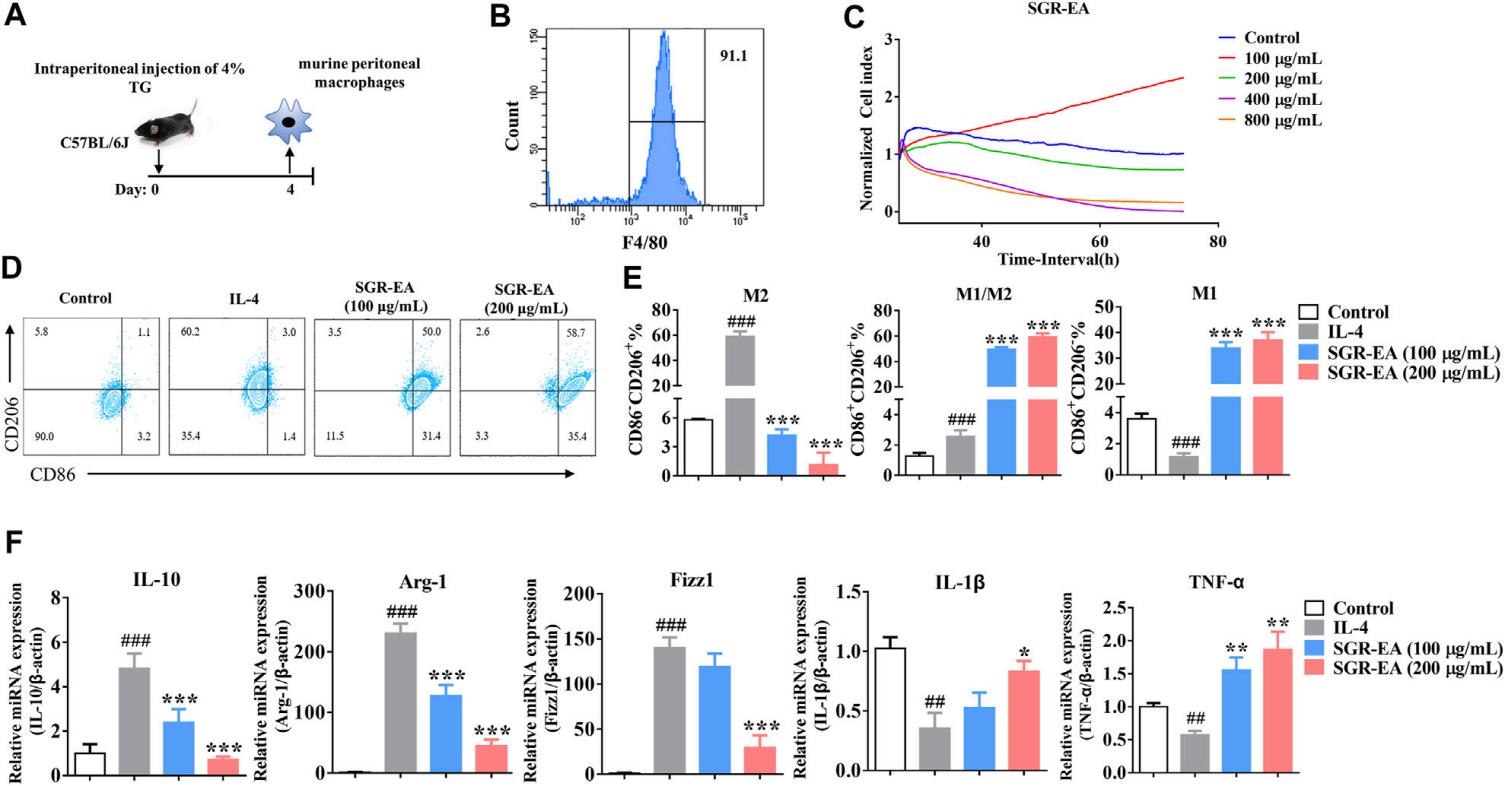
SGR-EA resets the tumor-related M2 macrophages to the M1 phenotype *in vitro*. **(A,B)** Schematic diagram of murine peritoneal macrophage extraction and purity identification. **(C)** Real-time cellular analysis (RTCA) safe concentration range of SGR-EA. **(D,E)** Percentages of M1 and M2 in the macrophages were detected by flow cytometry. **(F)** Expression levels of IL-10, Arg-1, Fizz1, IL-1β, and TNF-α in the macrophages were detected by qRT-PCR. Data are expressed as the mean ± SD. ^##^ *p* < 0.01 and ^###^ *p* < 0.001 versus control. **p < 0.01 and ***p < 0.001 versus model (IL-4).

To study the effect of SGR-EA on macrophage polarization, murine PMs were obtained with a purity of over 90%. Our study showed that there was no significant toxicity at macrophage-sensing SGR-EA concentrations less than or equal to 200 μg/ml (Figures 4A–C). The M2 activator IL-4 induced high expressions of CD206, arginase 1, IL-10, Fizz1, and SGR-EA in macrophages to inhibit the expression of M2 associated in a dose-dependent manner and promoted the expression of M1-associated markers such as CD86, IL-1β, and TNF-α (Figures 4D–F).

### *Smilax glabra* Roxb-Ethyl Acetate Functions Through a Hypoxia-Inducible Factor Signaling Pathway

**FIGURE 5:**
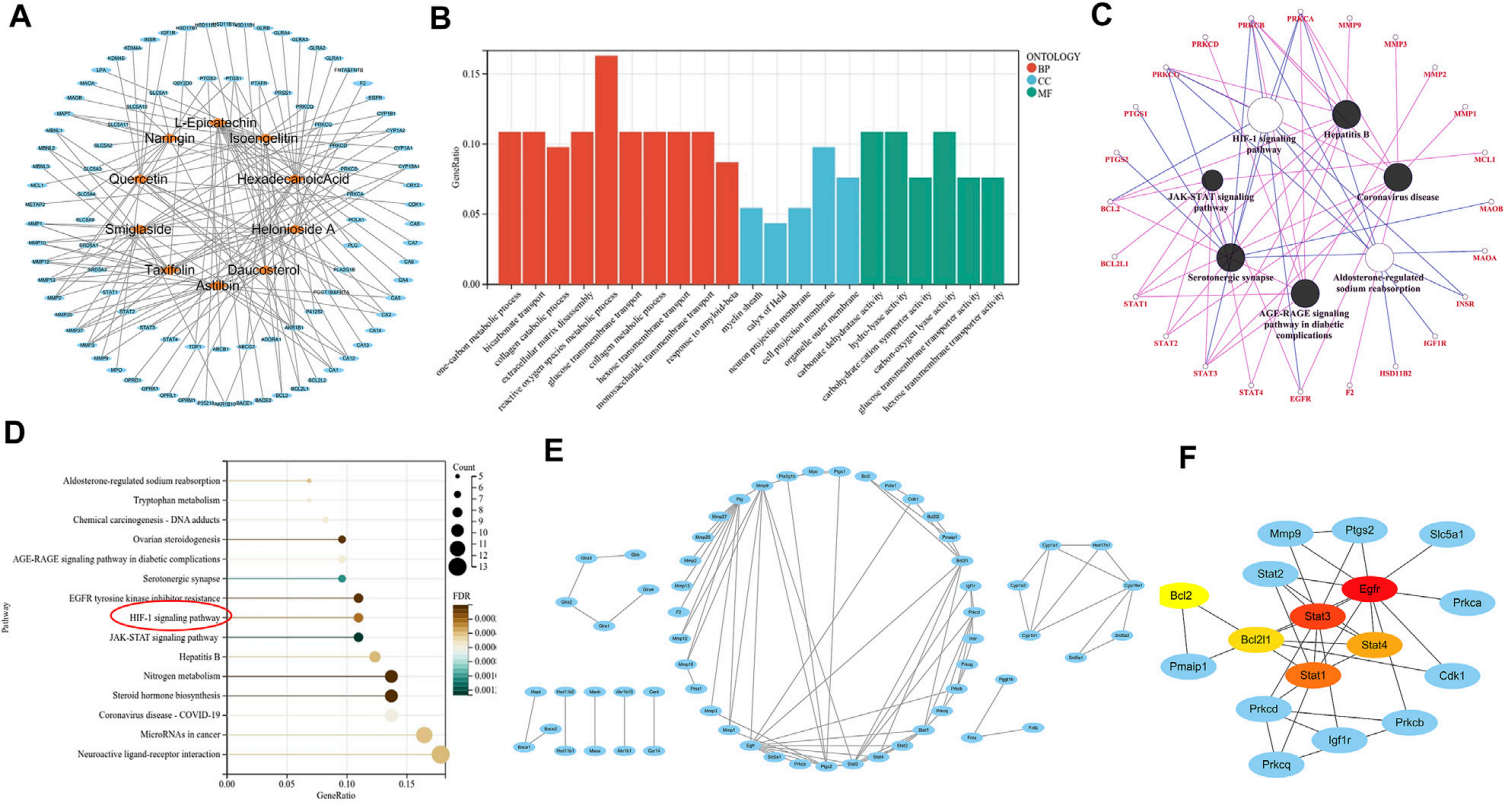
Pharmacologic analysis showed that SGR-EA functions through the HIF-1 signaling pathway. **(A)** SGR-EA drug–target relationship diagram. **(B)**. For the GO analysis of the SGR-EA target, red represents BP, blue represents CC, and green represents MF. **(C,D)** ClueGO and KEGG pathway enrichment analyses of the SGR-EA targets revealed that the HIF-1 signaling pathway may play an important role. **(E,F)** Protein interaction network analysis was performed on SGR-EA targets, and Cytohubba was used to screen Stat3 as the core of the protein network. The redder the color, the stronger the gene association was.

The SwissTargetPrediction platform was used to predict the target sites of 10 active compounds. After removing the repeated targets, 96 potential targets of the main active compounds in the ethyl acetate layer of SGR-EA were identified. Subsequently, Cytoscape was used to construct the drug network (Figure 5A). To further analyze the mechanism of action of SGR-EA in the treatment of breast cancer, we used the R package for GO and KEGG analyses of the target genes. In total, 659 GO items were screened, including 511 biological process (BP) items, 29 cell component (CC), and 119 molecular function (MF) items. The top 15 items of BP, top five items of CC, and top five items of MF were selected for a visual analysis according to the order of P from small to large, as shown in Figure 5B. The results showed that the biological processes involved in these core targets mainly included one-carbon metabolic process, bicarbonate transport, and collagen catabolic processes. A total of 103 pathways (*p* < 0.05) were obtained from the KEGG enrichment analysis, sorted in an ascending order of P, and the first 15 pathways were selected for visual analysis (Figure 5D). Simultaneously, we used ClueGO to analyze SGR-EA targets (Figure 5C). These results indicate that SGR-EA may exert anti-breast cancer effects through the HIF-1 signaling pathway. The protein interaction network of the target was further analyzed using STRING and visualized using Cytoscape (Figure 5E). Finally, the core genes were screened by Cytohubba. The results showed that Stat3 was at the core of the network (Figure 5F).

### *Smilax glabra* Roxb–Ethyl Acetate Regulation of Macrophage M2 Polarization Through a Hypoxia-Inducible Factor Signaling Pathway

**FIGURE 6:**
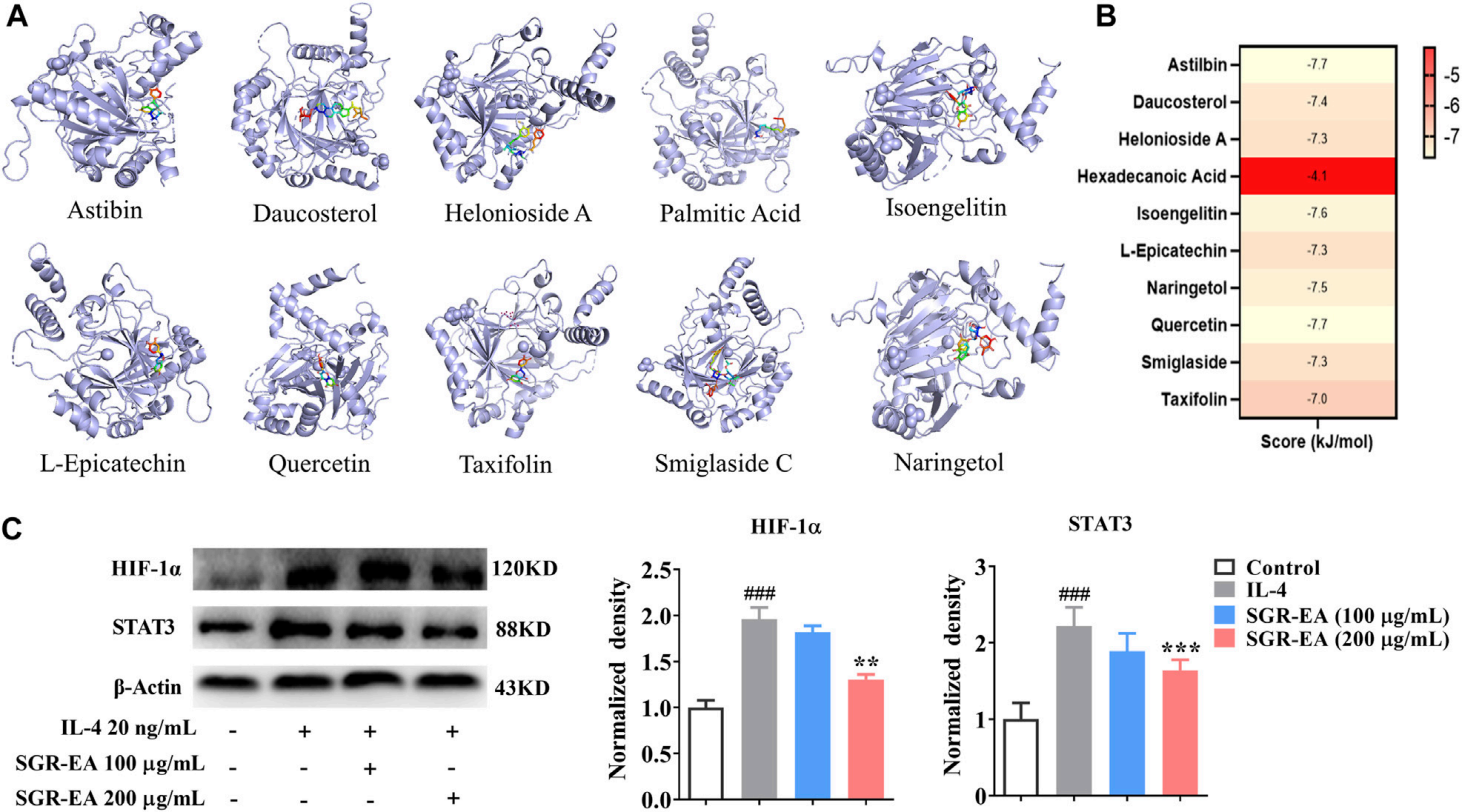
*In vitro* experiments verify that SGR-EA regulates macrophage M2 polarization through the HIF-1 signaling pathway. **(A)** Docking mode between core active components and the target protein. **(B)** Molecular docking results for all compounds benchmarking pleconaril. **(C)** Levels of HIF-1α and STAT3 proteins as determined by Western blotting. ^###^ *p* < 0.001 versus control. **p < 0.01 and ***p < 0.001 versus model (IL-4).

The 10 active components were screened by molecular docking using HIF-1. The compounds were docked to the protein active pocket, and their affinities are shown in Figure 6A. The docking results of each compound are shown in Figure 6B. Furthermore, we verified the effect of SGR-EA on the HIF-1 signaling pathway protein expression and found that SGR-EA significantly downregulated HIF-1α and STAT3 expressions in a dose-dependent manner (Figure 6C) *in vitro*.

## Discussion

SGR has a long history of treating various diseases, such as psoriasis, through its anti-inflammatory and immunomodulatory effects (Hua et al., 2018; Xu et al., 2022). The main components of SGR have been reported to inhibit tumor progression by inducing apoptosis and inhibiting cell proliferation (Samarakoon et al., 2012; Gao et al., 2018). However, few studies have shown that SGR inhibits tumor progression from the TAM perspective. This study is the first to confirm the anti-tumor effect of SGR in the MMTV-PyMT mouse model, providing an anti-cancer mechanism from the perspective of reversing the M2 polarization of TAMs.

The formation of an immunosuppressive microenvironment is closely associated with the progression of breast cancer. Therefore, remodeling the tumor immune microenvironment to improve the anti-tumor immune response is of great significance in inhibiting the progression of breast cancer (Bates et al., 2018; Byrne et al., 2020). Evaluation of the TME *in vivo* and IL-4 stimulation of macrophages *in vitro* suggested that SGR-EA delayed tumor progression by reversing the M2 polarization of TAMs and was associated with the inhibition of the HIF-1 signaling pathway.

It is well known that TAMs play an important role in the TME (Deberardinis, 2020). Currently, it is widely believed that M1 macrophages can exert anti-tumor effects in different ways, but in the process of tumor progression, tumor cells can directly or indirectly cause macrophages to develop into the M2 phenotype. In this study, we found that TAMs in the TME were reset by SGR-EA, from a tumor-promoting M2 phenotype to a tumor-suppressing M1 phenotype. Moreover, TAMs have been shown to release various cytokines and signaling molecules, such as the vascular endothelial growth factor (VEGF), to promote the formation and metastasis of vascular abnormalities, whereas M2-derived arginase 1 directly destroys the proliferation and activation of T cells to inhibit tumor immunity.

Furthermore, we analyzed the changes in T cells in the TME of the mice after drug administration. CD4^+^ T cells are important immune cells in the human immune system and are mainly expressed in T helper cells. Studies have found that in tumor immunity, CD4^+^ T cells can activate CD8^+^ T cells through various mechanisms after being activated to differentiate into cytotoxic T-lymphocytes, while maintaining and enhancing their anti-tumor response. However, even in the absence of CD8^+^ T cells, CD4^+^ T cells can directly kill tumor cells *via* the IFN-γ mechanism (Binnewies et al., 2019). Our experimental results showed that the proportion of CD4^+^T cells and CD8^+^ T cells in tumors increased after drug administration, but the difference was not statistically significant. In summary, our results provide new insights into the anti-tumor mechanisms of SGR, which plays an anti-tumor role by reversing the polarization of tumor-associated M1 to M2 macrophages and promoting the recruitment of CD4^+^ and CD8^+^ T cells in TAMs.

HIF-1α is an important nuclear transcription factor that regulates the cell hypoxia response, and its up-regulation is closely related to M2 polarization of TAMs (Movafagh et al., 2015). Under hypoxic conditions, HIF-1 signaling is activated, causing the expression of the genes regulated by it, such as VEGF and IL-6, to change. This changes the phenotype and metabolism of the tumor-associated macrophages from a pro-inflammatory (so-called M1-like) to an anti-inflammatory (so-called M2-like) state (Capece et al., 2013; Vitale et al., 2019). Macrophages are attracted to the hypoxic region of the tumor and gradually transform into M2 macrophages, further aggravating immunosuppression (Duechler et al., 2014). The flow cytometric analysis of tumors in mice showed that TAMs were reversed from the M2 to M1 state by SGR-EA after drug administration, further exerting anti-tumor effects. Moreover, IL-4 was used to construct an M2 polarization model *in vitro*. SGR-EA was found to reverse the M2 polarization process, and HIF-1 was verified as a possible target. However, there are still some shortcomings in this study. We only studied the anti-tumor effect of SGR-EA through TAMs’ regulation of the tumor microenvironment and did not clarify the role of specific components in SGR-EA. How SGR-EA plays a role through the STAT3/HIF1 signaling pathway is not discussed in depth, which is also one of the limitations to this study.

**FIGURE 7:**
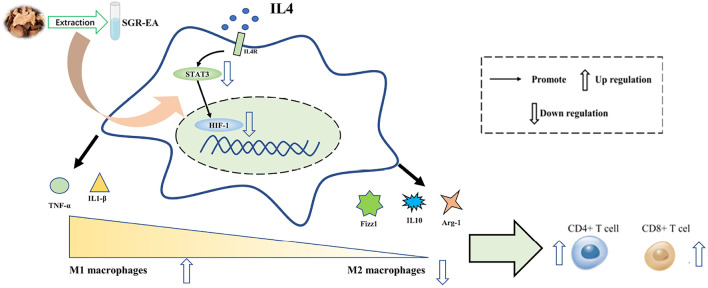
Mechanism Hypothesis Diagram.

In conclusion, SGR-EA has an obvious anti-breast cancer effect, specifically by inhibiting the activation of the HIF-1 signaling pathway of tumor-associated macrophages, reversing the M2 polarization of tumor-associated macrophages, and regulating the tumor immune microenvironment. “In conclusion, SGR-EA has an obvious anti-breast cancer effect, mainly by inhibiting the activation of HIF-1 signaling pathway of tumor-associated macrophages, reversing the M2 polarization of tumor-associated macrophages, and regulating the tumor immune microenvironment (Figure 7).”

## Data Availability

The original contributions presented in the study are included in the article/supplementary material; further inquiries can be directed to the corresponding authors.
